# Adverse Childhood Experiences and Substance Use: The Mediating Role of Executive Function Deficits

**DOI:** 10.1002/jad.70192

**Published:** 2026-06-04

**Authors:** Mahsa P. Yousefkhani, Xiafei Wang, Sara A. Vasilenko

**Affiliations:** ^1^ Department of Human Development and Family Science Syracuse University Syracuse New York USA; ^2^ College of Social Work University of Kentucky Lexington Kentucky USA

**Keywords:** ABCD study, adolescence, adverse childhood experiences, executive function, longitudinal, substance use

## Abstract

**Introduction:**

While adverse childhood experiences (ACEs) are robust risk factors for adolescent substance use, the prospective neurocognitive pathways linking both cumulative and specific adversities to substance use remain understudied. This study tested a vulnerability model by examining whether executive function (EF) deficits mediate the link between cumulative and specific ACEs and substance use, and whether this pathway differs for deprivation‐ versus threat‐based adversities.

**Methods:**

This longitudinal study used parent‐ and youth‐report data from the US Adolescent Brain Cognitive Development (ABCD) Study (*N* = 4731; M_age_ at baseline = 9.9 years; 52.3% male; 56.5% White, 13.8% Hispanic, 13.1% Black). We tested mediation models with temporally ordered assessments of ACEs (ages 9–11), parent‐reported EF deficits (12–13), and subsequent substance use (13–14).

**Results:**

The indirect effect of cumulative ACEs on substance use through EF deficits was significant, accounting for 10% of the total effect. Deprivation‐based ACEs (material hardship and emotional neglect) were fully mediated by EF. Several threat‐based adversities (e.g., household violence, bullying) also showed full mediation. In contrast, sexual abuse demonstrated a significant direct effect but no significant indirect effect through EF.

**Conclusions:**

EF deficits represent a significant pathway from cumulative ACEs to adolescent substance use, supporting a vulnerability model. This pathway appears robust for deprivation‐based and many chaotic threat‐based adversities, but sexual abuse is an exception that likely operates through non‐EF, trauma‐specific mechanisms. Prevention efforts should be multifaceted, incorporating EF‐focused interventions for at‐risk youth while prioritizing trauma‐focused care for survivors of sexual abuse.

Adolescent substance use remains a pressing public health concern (Harris and Weitzman 2024). Early initiation of alcohol, cannabis, nicotine, and other substances can cascade into academic failure (Wills et al. 2016), mental health problems (Boden et al. 2020), poorer physical health (Hamidullah et al. 2020), and substance use disorders in adulthood (Jordan and Andersen 2017; Volkow et al. 2021). Adverse childhood experiences (ACEs), including abuse, neglect, and household dysfunction, are a risk factor for substance misuse (Grummitt et al. 2022), estimated to account for 13%–29% of drug use and 5%–14% of smoking cases in the United States (Grummitt et al. 2022; Hughes et al. 2017). Foundational work demonstrated that individuals with four or more ACEs have a 4‐ to 12‐fold increased risk for alcoholism and drug abuse (Felitti et al. 1998), and subsequent research confirms adolescents with higher ACE scores initiate substance use earlier and progress more quickly to hazardous use (Leza et al. 2021; Ports et al. 2019). While the association is well‐established, the specific neurocognitive mechanisms explaining how early adversity translates into substance use remain an area of active investigation, with most studies employing cross‐sectional designs. To clarify these mechanisms prospectively, this study leverages longitudinal Adolescent Brain Cognitive Development (ABCD) Study data to examine executive function (EF) as a pathway linking cumulative and domain‐specific childhood adversities to early adolescent substance use initiation.

Vulnerability models suggest neurocognitive liabilities, such as EF deficits resulting from early adversity, act as premorbid risk factors creating a predisposition for addiction (Luciana 2020). EF is a multifaceted construct of higher‐order cognitive processes, including inhibitory control, working memory, and cognitive flexibility, that support self‐regulation and goal‐directed behavior (Diamond 2013; Miyake et al. 2000). A recent meta‐analysis concluded early adversity significantly reduces lifespan cognitive control, emphasizing EF as a mediator of ACEs' effects on later health and behavioral outcomes (Rahapsari and Levita 2025). Weaker EF skills have been linked to impulsivity, risk‐taking, and greater substance use across adolescence and young adulthood (Casey and Jones 2010; Luciana 2020), and longitudinal studies consistently show low preschool behavioral control predicts earlier and more problematic substance use by early adulthood (Dick et al. 2013; McGue et al. 2001; Peeters et al. 2015; Wilson et al. 2021). Importantly, ACEs are associated with deficits in daily‐life EF (Ifroh and Gai 2025; Trossman et al. 2022, 2021), with these deficits being one of the most frequently reported neurocognitive consequences of childhood maltreatment (Kavanaugh et al. 2017). This vulnerability is particularly pronounced during adolescence, a period characterized by heightened risk‐taking and an imbalance between a rapidly maturing reward system and a still‐developing prefrontal cortex responsible for executive control (Casey and Jones 2010; Spear 2000; Sturman and Moghaddam 2011). Neurobiological models explain chronic stress from adversity disrupts the maturation of these prefrontal and frontoparietal networks, compromising the neural circuits subserving EF (McLaughlin and Sheridan 2016; Teicher et al. 2016; Trossman et al. 2021).

A growing body of research provides direct support for a mediational model where executive dysfunction links adversity to substance misuse. For example, Ifroh and Gai (2025) found EF deficits partially mediated the relationship between ACEs and health‐risk behaviors, including smoking and vaping, in Indonesian adolescents. This pathway has also been established neurobiologically, with trauma‐affected brain networks predicting future high‐risk drinking via their impact on executive dysfunction (Silveira et al. 2020). Furthermore, Trossman et al. (2022) identified a crucial methodological nuance, finding that self‐reported global executive dysfunction, capturing EF difficulties in daily life, mediated the link between adversity and poor mental health, whereas performance on discrete, lab‐based core executive skills did not. However, a competing explanation exists for the relationship between EF and substance use. Neurotoxicity models propose that substance use is the causal agent that leads to subsequent changes in normal brain development, resulting in EF deficits and a loss of control (Castellanos‐Ryan et al. 2017), which subsequently increases the propensity to seek substances (Scott et al. 2007; Squeglia et al. 2009; Wilens et al. 2011). Much of the existing research examining the ACEs‐EF‐substance use pathway, however, has relied on cross‐sectional designs, making it difficult to establish which mechanism is operating in the EF‐substance use link.

Most studies examining pathways from early ACEs to future health outcomes utilize the cumulative risk model, which treats all adversities as equally impactful by summing them into a single score (Evans et al. 2013; Felitti et al. 1998). However, this approach is criticized for ignoring how distinct experiences may influence development through different mechanisms (McLaughlin and Sheridan 2016). Alternatively, the Dimensional Model of Adversity and Psychopathology (DMAP) conceptualizes adversities along two core dimensions: threat, defined by the presence of harm (e.g., physical abuse), and deprivation, defined by the absence of expected cognitive and social inputs (e.g., emotional neglect; McLaughlin and Sheridan 2016; Sheridan and McLaughlin 2014). DMAP posits these dimensions have partially distinct downstream consequences. Specifically, it predicts deprivation is more likely to disrupt the development of prefrontal cognitive systems supporting EF, whereas threat is more likely to alter emotion processing and fear learning circuits centered on the amygdala (Sheridan and McLaughlin 2014). A systematic review by Johnson et al. (2021) found that while both threat and deprivation were associated with reduced EF, the association was significantly stronger for deprivation. Notably, while DMAP is sometimes operationalized by aggregating adversities into composite threat and deprivation scores (e.g., Miller et al. 2018), McLaughlin et al. (2021) emphasize that because adversities naturally co‐occur, ACEs should be modeled simultaneously to isolate unique contributions and avoid misattributing the effects of one dimension to another. Drawing on this recommendation, the present study models all 13 ACEs simultaneously and interprets the resulting pathways through the DMAP framework.

Despite this foundation, several gaps remain in the literature. Much of the existing research relies on cross‐sectional designs, limiting causal inference, or small clinical samples, limiting generalizability (Luciana 2020; Trossman et al. 2021). Furthermore, most studies focus on cumulative ACE scores without differentiating specific domains, seldom utilize large, representative cohorts, and rarely examine whether EF mediation persists after controlling for co‑occurring ACEs (Johnson et al. 2021). Finally, EF mediation magnitude remains unclear because earlier work often omitted relevant covariates such as socio‑economic status and neurodevelopmental disorders (Rahapsari and Levita 2025; Johnson et al. 2021).

## 1 Current Study

The present study addresses the aforementioned gaps using data from the ABCD Study, a prospective cohort following US youth from ages 9 to 10 (Karcher and Barch 2021). We utilize data tracking ACEs (reported at baseline and year 1), EF deficits (measured at year 3), and substance use (assessed at year 4 when participants are 13–14 years old). This longitudinal design allows us to test whether ACEs and EF predict subsequent substance use independent of pre‐existing substance use, thus providing stronger support for the vulnerability model over a competing neurotoxicity account. The present analysis is novel in its specific testing of EF as a mediator of both cumulative and domain‐specific ACEs on substance use across these specific developmental time points.

Hypothesis 1 Informed by the cumulative risk model, we hypothesize that a higher number of ACEs is associated with substance misuse after controlling for other co‐occurring ACEs.

Exploratory aim: We have an exploratory aim to examine which specific ACEs uniquely predict substance use after accounting for the effects of all other co‐occurring ACEs.

Hypothesis 2 Informed by the vulnerability model of addiction, we hypothesize that ACEs will predict EF deficits, which in turn will predict substance use. Specifically, we expect EF deficits to partially mediate this pathway.

Hypothesis 3 Informed by the DMAP framework, we hypothesize that this mediation effect will be domain‐specific, occurring only for deprivation‐based ACEs and not for threat‐based ACEs.

## 2 Methods

### 2.1 Data and Sample

Data were drawn from the ABCD Study, a prospective cohort following approximately 11,868 youth from ages 9–10 for at least 10 years into late adolescence and early adulthood (Karcher and Barch 2021). A full description of the ABCD Study design and measures is available elsewhere (Barch et al. 2021). Data were accessed from the ABCD Study Data Release 5.0.

The initial cohort included 11,868 participants with complete data on ACEs at baseline and year 1. The final analytic sample comprised 4731 participants (M_age_ = 9.9 years; 52.3% male) who were racially and ethnically diverse (56.5% White, 13.8% Hispanic/Latino, 13.1% Black). Comprehensive descriptive statistics for all study variables are provided in Table 1. All study procedures were approved by the institutional review boards at each of the 21 research sites, and informed consent was obtained from parents and assent from the children.

**Table 1:** Sociodemographic characteristics and key study variables of the analytic sample (*N* = 4731).

Variable	*N* (Valid)	Mean (SD) or %
Sample characteristics (baseline)		
Parental age	4708	40.39 (6.68)
Parental education^a^	4724	16.83 (2.65)
Income‐to‐needs ratio^b^	4339	4.09 (2.84)
Adolescent sex	4730	
Male (reference)		52.3%
Female		47.7%
Adolescent race/ethnicity	4669	
White (reference)		56.5%
Black		13.1%
Hispanic/Latino		13.8%
Asian		5.5%
Native American/Alaska Native		1.8%
Other/Multi‐Racial		9.2%
ADHD (baseline) clinical range (T ≥ 70)^c^		
Non‐clinical range	4605	97.4%
Clinical range	125	2.6%
Prior substance use (year 3)	4659	1.8%
Predictor: Adverse childhood experiences (baseline & year 1)		
Cumulative ACEs (0−9)	4731	2.23 (1.56)
Specific ACEs (% endorsed)		
Household mental illness	4521	59.0%
Household alcohol misuse	4590	45.6%
Community violence	4638	33.2%
Traumatic grief	4632	21.8%
Material hardship	4710	19.4%
Bullying	4731	13.8%
Parental divorce/separation	4711	9.4%
Emotional neglect	4730	9.0%
Household criminal justice involvement	4632	7.4%
Household violence	4589	6.8%
Crime victimization	4636	1.3%
Physical abuse	4589	0.9%
Sexual abuse	4589	0.9%
Mediator: Executive function (year 3)		
Executive function deficits (centered)^d^	4588	0.05 (8.39)
Outcome: Substance use (year 4)		
Any substance use (past year)^e^	4731	6.4%

*Note:* The number of valid responses (*N*) and corresponding percentages for each variable may differ slightly from the total sample size due to item‐level missing data.

Abbreviations: ACEs = adverse childhood experiences; ADHD = attention‐deficit/hyperactivity disorder; SD = standard deviation.

^a^ Parental education reflects the highest level attained by either parent on a 0–21 scale.

^b^ Income‐to‐needs ratio (household income relative to the federal poverty threshold for household size); higher values reflect greater financial resources.

^c^ Clinical Range (T ≥ 70) coded as 0 = no, 1 = yes based on the Child Behavior Checklist.

^d^ Summary score from the Barkley Deficits in Executive Functioning Scale–Children and Adolescents (BDEFS‐CA) at Year 3, centered at the sample mean. Higher scores indicate greater EF deficits.

^e^ Binary variable indicating any substance use initiation reported at the 4‐year follow‐up.

### 2.2 Measures

#### 2.2.1 Substance Use

Substance use was assessed at the 4‐year follow‐up (ages 13–14) using a modified, computerized Timeline Followback (TLFB) interview, a well‐validated, interviewer‐administered tool (Lisdahl et al. 2018; Sobel and Sobell 1996). The TLFB prompted participants to report on substance use “since we last saw you,” capturing use during the year between the 3‐ and 4‐year follow‐ups. The interview assessed use of alcohol (full drinks), nicotine (combustible cigarettes, e‐cigarettes, cigars, chew, hookah), and cannabis (marijuana, blunts, edibles). A binary composite outcome represented any new substance use initiation. Participants were coded as “1” if they endorsed using any of the nine substance types for the first time during the past year, and “0” if they reported no use.

#### 2.2.2 ACEs

Thirteen indicators of ACEs were assessed using parent‐report and youth self‐report data from the baseline and Year‐1 follow‐up assessments. The selection of indicators was guided by the original ACEs framework (Felitti et al. 1998) and subsequent expansions (Nagata et al. 2023). Data were drawn from instruments including the Kiddie‐Schedule for Affective Disorders and Schizophrenia (KSADS). The 13 dichotomous (0 = no, 1 = yes) indicators included: physical abuse, sexual abuse, emotional neglect, parental divorce, household alcohol misuse, household mental illness, household violence, household criminal justice involvement, community violence, material hardship, bullying, crime victimization, and traumatic grief. For indicators assessed at both waves (community violence, emotional neglect, household violence), a composite “ever‐exposed” variable was created. A cumulative ACEs score was then calculated by summing the 13 indicators (range: 0–13). A full description of the operationalization of the specific ACE indicators can be found elsewhere (Wang et al. 2025).

#### 2.2.3 EF

EF deficits were measured at year‐3 (ages 12–13) using the caregiver‐report Barkley Deficits in Executive Functioning Scale–Children and Adolescents (BDEFS‐CA; Barkley 2012). This scale assesses daily‐life EF challenges across domains such as self‐restraint, time management, and emotional self‐regulation on a 4‐point scale (1 = *never or rarely* to 4 = *very often*). A total summary score was calculated by summing the items, where higher scores indicate greater deficits. The scale has demonstrated excellent internal consistency in the ABCD cohort (Cronbach's *α* = 0.93; Peisch et al. 2024).

#### 2.2.4 Control Variables

We included demographic and socioeconomic covariates in all models, assessed at baseline via parent report, including child's sex assigned at birth (0 = male, 1 = female), parental age and education, binary indicators of race/ethnicity (White as the reference group), and income‐to‐needs ratio (ITN) (M. R. Gonzalez et al. 2020; Rakesh et al. 2022). Parental education was operationalized as the highest level of schooling completed by either parent on a continuous scale ranging from 0 (no formal education) to 21 (doctoral degree). The ITN was calculated by dividing the median value of each household's reported income bracket by the 2017 US Federal Poverty Guidelines for the corresponding household size, with higher values reflecting greater financial resources. Although ITN and the material hardship ACE indicator are conceptually related, they remain theoretically distinct. ITN captures the household's structural socioeconomic position, whereas material hardship captures the experiential adversity of deprivation (e.g., inability to afford basic needs). Including ITN as a covariate, therefore, enables us to isolate the unique contribution of experiencing material hardship beyond the household's background socioeconomic context, consistent with distinctions in the ACE literature (Gershoff et al. 2007; Nagata et al. 2023).

To isolate the unique effects of ACEs and test our primary hypotheses, we included two additional covariates. First, baseline symptoms of attention‐deficit/hyperactivity disorder (ADHD) were included in the model to account for pre‐existing neurodevelopmental vulnerabilities and capture the well‐documented externalizing pathway to early substance use (Fava et al. 2019; Groenman et al. 2017; Howard et al. 2020). ADHD was assessed using the parent‐reported Child Behavior Checklist (CBCL; Achenbach and Rescorla 2001) and operationalized as a binary variable indicating whether a participant's score on the DSM‐5 oriented ADHD scale fell in the clinical range (*T*‐score ≥ 70). Second, to test the vulnerability model against a competing neurotoxicity explanation, we controlled for prior substance use. This was operationalized as a binary composite variable for any use of nine substance types in the year prior, created from the TLFB interview at the 3‐year follow‐up.

## 3 Results

### 3.1 Analytic Strategy

All statistical analyses were conducted in SPSS (Version 29). Missing data on the Year‐4 substance use outcome primarily reflected the rolling structure of the ABCD Study's data releases rather than traditional attrition, as Year‐4 follow‐up was ongoing during Release 5.0. Therefore, listwise deletion restricted the analytic sample to participants with complete primary outcome data (*N* = 4731 from the baseline *N* = 11,868). Within this sample, residual missingness on covariates was handled via default listwise deletion in the PROCESS macro (Hayes 2022), producing minor sample size variation across models.

Preliminary analyses first assessed the direct effect of the cumulative ACE score on substance use via logistic regression, controlling for demographic covariates, baseline ADHD, and prior substance use. Next, a series of logistic regressions assessed the unique direct effect of each of the 13 specific ACEs. To isolate each target ACE's unique contribution while accounting for adversity clustering (Dong et al. 2004; Finkelhor et al. 2007), these specific‐ACE models controlled for the standard covariates plus the remaining 12 ACEs (hereafter referred to as “co‐occurring ACEs”).

Primary mediation hypotheses were tested using the PROCESS macro for SPSS (Model 4; Hayes 2022). Indirect effects were tested with 5000 bootstrap samples, with significance determined by a 95% confidence interval (CI) not containing zero. We first estimated a mediation model for the cumulative ACEs score to test the overall pathway. Subsequently, we estimated separate mediation models for each of the 13 specific ACEs, controlling for standard covariates and co‐occurring ACEs, to isolate unique mediational pathways.

### 3.2 Sensitivity Analyses

To examine whether participants in the analytic sample differed from those without Year‐4 outcome data, we compared the two groups on cumulative and specific ACEs. Mean cumulative ACE exposure was slightly higher among those without Year‐4 data (M = 2.36, SD = 1.64) than those included (M = 2.23, SD = 1.56), *t*(11,866) = 4.40, *p* < 0.001, but the difference was negligible (Cohen's *d* ≈ 0.08). Six specific ACEs showed statistically significant differences (household mental illness, household violence, household criminal justice system (CJS) involvement, community violence, material hardship, and bullying), but all effect sizes were small (largest *φ* = 0.053). Given the design‐driven nature of missingness and negligible group differences, listwise deletion was judged appropriate.

### 3.3 Regression Analyses

First, to test the overall dose‐response relationship (Hypothesis 1), a logistic regression model controlling for demographics, baseline ADHD, and prior substance use confirmed that the cumulative ACE score was a significant predictor of substance use at the 4‐year follow‐up (*b* = 0.170, OR = 1.185) (Table 2).

**Table 2:** Summary of logistic regression analyses predicting substance use from specific ACEs and mediation models for specific ACEs predicting substance use via executive function.

	(Regression model)				(Mediation model)				
	*b*	SE	OR	95% CI	Path a (*b*)	Path b (*b*)	Direct effect (b)	Indirect effect (*b*)	95% CI for indirect effect
Cumulative ACE	**0.170** ***	**0.043**	**1.185**	**[1.09, 1.29]**	**1.144** ***	**0.016** *	**0.162** ***	**0.018**	**[0.002, 0.034]**
Physical abuse	−0.854	0.804	0.43	[0.09, 2.06]	−0.38	0.019*	−1.29	−0.007	[−0.080, 0.064]
Sexual abuse	**1.048** *	**0.51**	**2.85**	**[1.05, 7.73]**	−0.84	0.019*	0.95	−0.016	[−0.084, 0.049]
Emotional neglect	−0.263	0.269	1.00	[0.59, 1.68]	1.76***	0.019*	−0.29	**0.033**	**[0.005, 0.071]**
Parental divorce	0.283	0.215	1.33	[0.87, 2.02]	0.058	0.019*	0.266	0.001	[−0.018, 0.021]
Hshld. Alcohol use	0.264	0.148	1.30	[0.98, 1.74]	0.48	0.019*	0.26	0.009	[−0.001, 0.025]
Hshld. Mental illness	0.021	0.154	1.02	[0.76, 1.38]	1.42***	0.019*	−0.014	**0.027**	**[0.005, 0.053]**
Hshld. Violence	−0.002	0.266	1.00	[0.59, 1.68]	1.63**	0.019*	−0.015	**0.031**	**[0.003, 0.072]**
Hshld. CJS	**0.480** *	**0.239**	**1.62**	**[1.01, 2.59]**	1.47**	0.019*	0.39	**0.028**	**[0.001, 0.066]**
Community violence	−0.05	0.167	0.95	[0.69, 1.32]	0.756*	0.019*	−0.054	**0.014**	**[0.001, 0.034]**
Material hardship	0.054	0.197	1.06	[0.72, 1.55]	2.09***	0.019*	0.020	**0.040**	**[0.007, 0.078]**
Bullying	0.292	0.187	1.34	[0.93, 1.93]	3.07***	0.019*	0.275	**0.058**	**[0.011, 0.110]**
Crime victimization	0.17	0.564	1.19	[0.39, 3.58]	3.06*	0.019*	0.163	0.058	[−0.004, 0.153]
Traumatic grief	0.216	0.158	1.24	[0.91, 1.69]	0.677*	0.019*	0.225	0.013	[−0.000, 0.032]

*Note:* For regression model: *b* = unstandardized logistic regression coefficient. For mediation model: *b* = unstandardized coefficient. Path a = effect of ACE on executive function (EF). Path c' = direct effect of ACE on substance use, controlling for EF. Indirect effect = effect of ACE on substance use via EF. All models predict substance use at 4‐year follow‐up. All models control for income‐to‐needs, adolescent sex, race/ethnicity, parental education, parental age, baseline ADHD, prior substance use at year 3, and the other 12 ACEs. Significant indirect effects (and their 95% bootstrap CIs) are shown in bold.

Abbreviations: CI = confidence interval; OR = odds ratio; SE = standard error.

* *p* < 0.05

** *p* < 0.01

*** *p* < 0.001.

To address our exploratory aim, we then conducted a series of logistic regression models to assess the unique relationship of each of the 13 specific ACEs, controlling for all other co‐occurring ACEs in addition to the standard covariates. From these fully adjusted models, two ACEs emerged as significant unique predictors: sexual abuse (OR = 2.85) and household criminal‐justice involvement (OR = 1.62) (Table 2).

### 3.4 Mediation Analysis: Cumulative ACEs

**Figure 1:**
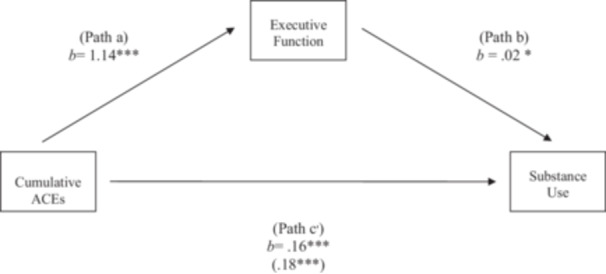
Path diagram of the mediation analysis linking cumulative adverse childhood experiences to adolescent substance use via executive function deficits. Values represent unstandardized regression coefficients (b). The coefficient on the path from cumulative ACEs to substance use represents the direct effect (Path c′), while the coefficient in parentheses represents the total effect (Path c). The model controls for the following covariates, all assessed at baseline unless otherwise noted: child sex, race/ethnicity, parental education, parental age, income‐to‐needs ratio, baseline ADHD clinical range (CBCL), and prior substance use (assessed at year‐3 follow‐up). ACEs = adverse childhood experiences; EF = executive function. *p < 0.05, **p < 0.01, ***p < 0.001.

To test the overall mediational pathway (Hypotheses 2 and 3), a mediation model was examined using the PROCESS macro (Model 4) with 5000 bootstrap samples (*N* = 4145). The results of the mediation analysis for cumulative ACEs are depicted in Figure 1. As predicted, all paths were statistically significant. The path from cumulative ACEs to EF deficits (Path a) was significant, indicating that each additional ACE was associated with greater EF deficits (*b* = 1.14, SE = 0.09, *p* < 0.001). In turn, greater EF deficits significantly increased the odds of substance use (Path b; *b* = 0.016, SE = 0.008, *p* = 0.045, OR = 1.02). The indirect effect of cumulative ACEs on substance use through EF deficits was significant (*b* = 0.018, OR = 1.02), indicating that each additional ACE increased the odds of substance use by approximately 2% through its impact on EF. The direct effect of ACEs also remained significant (Path c’; *b* = 0.16, *p* < 0.001, OR = 1.18), demonstrating partial mediation. Approximately 10% of the total effect of cumulative ACEs on substance use was transmitted through EF deficits.

### 3.5 Mediation Analyses: Specific ACEs

To test the hypothesis that specific adversity domains demonstrate unique indirect effects (Hypothesis 3), mediation models were estimated for each of the 13 ACEs, controlling for standard covariates and all other 12 ACEs. The results are presented in Table 2.

Seven ACEs demonstrated full mediation through EF deficits, with significant indirect effects but nonsignificant direct effects: material hardship (*b* = 0.040), household mental illness (*b* = 0.027), emotional neglect (*b* = 0.033), household violence (*b* = 0.031), community violence (*b* = 0.014), bullying (*b* = 0.058), and household CJS involvement (*b* = 0.028).

In contrast, six ACEs showed no significant direct or indirect associations with substance use when controlling for all covariates including co‐occurring ACEs. These included crime victimization, household alcohol use, traumatic grief, sexual abuse, physical abuse, and parental divorce. Notably, sexual abuse showed a significant direct effect in the regression model (OR = 2.85) but no significant indirect pathway through EF deficits (95% CI [−0.084, 0.049]).

## 4 Discussion

The present study examined whether EF deficits serve as a mechanism linking ACEs to early adolescent substance use, and whether this pathway differs for deprivation‐ versus threat‐based adversities while accounting for their co‐occurrence. Our analyses yielded four primary findings. First, consistent with the cumulative risk model and foundational research (Felitti et al. 1998; Hughes et al. 2017; Leza et al. 2021), higher cumulative ACE scores prospectively predicted an increased likelihood of substance use. Second, when controlling for co‐occurring adversities, the effects of most specific ACEs attenuated, with only sexual abuse and household CJS involvement retaining a unique, direct association with substance use. Third, EF deficits partially mediated this cumulative relationship, accounting for approximately 10% of the total effect. Finally, the mediational pathway showed unexpected specificity: while deprivation‐based ACEs were fully mediated by EF as predicted by DMAP, several threat‐based adversities also showed full mediation, whereas sexual abuse showed an unmediated direct effect.

### 4.1 Sexual Abuse and Substance Use

Our exploratory analysis revealed that not all adversities carry equal risk. Once shared variance among all ACEs was accounted for, most individual effects became nonsignificant, leaving only sexual abuse and household CJS involvement as unique predictors. Our finding that sexual abuse remained a significant predictor converges with a diverse body of multivariable, long‐term prospective, genetically informed, and meta‐analytic literature identifying it as a particularly durable risk factor for substance misuse (Merrick et al. 2017; Fergusson et al. 2008, 2013; Grummitt et al. 2022; Sebalo et al. 2023). The robustness of this association likely reflects the distinct trauma profile of sexual abuse, characterized by shame, dissociation, and PTSD, which promotes substance use for affect regulation (Douglas et al. 2010; Grummitt et al. 2022). Although some research suggests this effect dissipates when accounting for cumulative adversity (Goldstein et al. 2013; Hoffmann and Jones 2022), such discrepancies often reflect methodological differences in sample age and outcome measurement. Our results demonstrate that sexual abuse is a distinctive predictor of subsequent adolescent substance use, even after rigorous statistical control. Notably, this effect emerged despite a very low endorsement rate in our sample (0.9%), underscoring the magnitude of the individual‐level impact of sexual abuse on early adolescent substance use.

### 4.2 Household CJS and Substance Use

Household CJS involvement demonstrated a similar robust association, aligning with research linking parental incarceration to adult substance use (Gjelsvik et al. 2013) and young adult illicit drug disorders (Gifford et al. 2019). Longitudinal work supports this, showing paternal incarceration predicts steeper trajectories of marijuana use (Roettger et al. 2011) and population‐based surveys associating incarceration with early initiation and dependence (L. Davis and Shlafer 2017). However, some evidence suggests these associations are method‐dependent and may attenuate when controlling for cumulative adversity, SES, and parental criminality (Jackson et al. 2021; Kinner et al. 2007; Murray et al. 2012), or operate indirectly through victimization and strain (Jin et al. 2025). While much of the literature focuses on adult outcomes or finds null effects in adolescence after adjustment, our study identifies parental CJS involvement as a significant predictor of substance use during mid‐adolescence, even after accounting for household substance misuse. Parental CJS involvement remaining a unique predictor likely reflects its nature as an adversity straddling deprivation and threat. Families affected by incarceration systematically experience elevated poverty, mental illness, and violence (Aaron and Dallaire 2010; Turney 2018; Wildeman 2009). Consistent with this clustering pattern, post hoc bivariate correlations within our sample revealed that household CJS involvement was most strongly associated with household violence (*r* = 0.24), material hardship (*r* = 0.19), and community violence (*r* = 0.15), three ACEs that independently demonstrated full EF‐mediated pathways to substance use. This suggests the direct effect of household CJS may partly reflect the cumulative impact of these co‐occurring adversities. Household CJS involvement thus appears to function as a marker of clustered environmental risk, which may help explain why it retains a direct effect on substance use beyond the EF pathway.

By controlling for ADHD, our models more cleanly isolate the contribution of adversity exposure by accounting for the documented externalizing pathway where pre‐existing neurodevelopmental risk confers heritable liability for early and heavier substance involvement (Fava et al. 2019; Groenman et al. 2017; Howard et al. 2020). Importantly, this covariate captures a clinical externalizing risk profile that is conceptually and operationally distinct from the daily‐life EF deficits tested as the mediator in our model. Under these conditions, the finding that only sexual abuse and household CJS involvement remained significant supports the argument that adversities do not carry equal incremental risk when modeled together (Halpern et al. 2018; Merrick et al. 2017). Ultimately, these results move the field beyond a simple cumulative‐risk narrative toward a more nuanced, mechanism‐informed account of which specific adversities drive youth substance use (Grummitt et al. 2022; Mulia et al. 2025; Ramírez et al. 2025).

### 4.3 Cumulative ACEs—EF—Substance Use

Our mediation model provided evidence for our second hypothesis. Cumulative ACEs (ages 9–11) were prospectively associated with greater daily‐life EF deficits (ages 12–13), which in turn predicted increased odds of substance use (ages 13–14), even after controlling for prior use. This finding supports a vulnerability model where neurocognitive liabilities act as premorbid risk factors (Khurana et al. 2013; Luciana 2020; Peeters et al. 2015), a biologically plausible pathway (De Bellis and Zisk 2014) consistent with meta‐analytic evidence linking adversity to EF deficits (Op den Kelder et al. 2018; Rahapsari and Levita 2025). The literature remains nuanced, however, with null findings in some samples (Trossman et al. 2022) and evidence suggesting lower EF more strongly predicts early or escalating use rather than later dependence (Gustavson et al. 2017; Kräplin et al. 2022), indicating the pathway's strength may be moderated by developmental period and outcome. Crucially, our prospective design, which controlled for prior substance use, provides stronger support for a developmental vulnerability perspective (McLaughlin and Sheridan 2016) over a neurotoxicity account, although reciprocal influences or shared risk factors remain plausible over longer developmental spans (R. Gonzalez et al. 2017; Meier et al. 2018).

The mediating role of EF accounted for only about 10% of the total association between cumulative ACEs and substance use, leaving a substantial direct effect. This aligns with the broader literature, where EF typically explains 10%−25% of the total effect, whereas broader self‐regulation constructs encompassing externalizing behaviors often account for much larger proportions (Grummitt et al. 2021; Cooper et al. 2023). This partial mediation suggests that while EF deficits are a meaningful pathway, the remaining direct effect likely represents a confluence of other powerful mechanisms, including trauma‐related emotion dysregulation leading to self‐medication (Brewer‐Smyth 2022; Garland et al. 2019), alterations in brain reward and stress circuits (Levis et al. 2022), and the development of externalizing symptoms (Fava et al. 2019).

### 4.4 Specific ACEs—EF—Substance Use

Our third hypothesis, predicting that EF would mediate substance use pathways for deprivation‐based but not threat‐based ACEs, was partially supported. As predicted by DMAP, deprivation‐based ACEs (emotional neglect, material hardship) were fully mediated by EF. This aligns with meta‐analytic and prospective research showing deprivation has a stronger negative association with EF and related frontoparietal structures than threat experiences do (Rahapsari and Levita 2025; Johnson et al. 2021; Sheridan et al. 2017). Conversely, several threat‐based (household/community violence, bullying) and mixed adversities (household mental illness, household CJS involvement) were also fully mediated by EF. While seemingly contradictory to a strict DMAP interpretation, this pattern aligns with emerging critiques emphasizing that real‐world adversities are complex, hybrid exposures high in unpredictability and chaos, rather than “pure” threats (Smith and Pollak 2021; McLaughlin et al. 2021). For instance, household mental illness and CJS involvement may undermine EF through caregiving disruption (Haskins 2016; Gueron‐Sela et al. 2018), while chronic threats like household violence and bullying may deplete executive resources through hypervigilance, HPA activation, and social unpredictability (McEwen and Morrison 2013; Medeiros et al. 2016). Our everyday EF measure, capturing “hot” EF in affectively charged contexts rather than “cool” EF of lab tasks (Metcalfe and Mischel 1999), may be particularly sensitive to these chaotic adversities, explaining why their unique effects were fully absorbed by the mediator.

This context also reconciles our findings with prior work where certain threat‐type ACEs, particularly sexual abuse, retain strong direct effects on substance use. Consistent with this literature (e.g., Merrick et al. 2017; Grummitt et al. 2022), sexual abuse showed a direct but no EF‐mediated path, suggesting it is an important exception that bypasses general EF and instead operates through trauma‐specific mechanisms like altered HPA axis regulation (Trautmann et al. 2018) or blunted stress reactivity (Al'Absi et al. 2021). Ultimately, our findings support a nuanced view where deprivation‐type adversities may most consistently impair EF, but threat‐based adversities high in unpredictability and chaos may also affect EF through stress‐mediated and resource‐depletion pathways.

The pattern of EF‐mediated effects also speaks to ongoing debates about the scope of the ACE construct itself. Adversities demonstrating these pathways to substance use were predominantly household‐level and community‐level exposures. Earlier foundational work in the ACE literature focused largely on personal abuse and household dysfunction, with community‐level adversities receiving limited attention. Subsequent framework expansions argue that community‐level adversities, such as community violence, bullying, and neighborhood deprivation, are highly significant exposures necessary to capture a holistic picture of childhood adversity and its developmental impact (Cronholm et al. 2015; Finkelhor et al. 2007). Our findings empirically support this expanded conceptualization by demonstrating that community‐level adversities also show significant EF‐mediated pathways to substance use.

### 4.5 Implications

The finding that daily‐life EF deficits appear to mediate the link between childhood adversity and substance use carries clinical and preventative implications. Because our measure captures malleable, everyday self‐regulation, it aligns with evidence that EF skills can be improved through ecologically grounded approaches like structured curricula and mindfulness (Diamond and Lee 2011; Zelazo and Carlson 2012). However, EF's modest mediation (~10%) and the substantial remaining direct effect suggest a multimodal prevention strategy is warranted. Specifically, for deprivation‐based adversities, EF interventions could be coupled with policies enhancing cognitive stimulation; for adversities high in unpredictability, like household violence, stabilizing caregiver functioning may be as important as direct EF training (Grummitt et al. 2022; Merrick et al. 2017). Furthermore, not all pathways are EF‐dependent. The direct effect of sexual abuse suggests trauma‐focused treatments (e.g., trauma‐focused CBT) should be a primary consideration for these youth (Cohen et al. 2017).

More broadly, the predominance of household‐ and community‐level exposures among ACEs showing EF‐mediated pathways underscores the social environment's substantial role in shaping adolescent risk (Hawkins et al. 1992; Lin et al. 2024). Accordingly, individual‐level EF interventions are likely most effective when embedded within wraparound services and multi‐level community supports that address the contexts where adversity unfolds (Turney 2018; J. R. Olson et al. 2021). This includes economic supports for families experiencing material hardship or CJS involvement, caregiver mental health services, and family‐ and community‐level violence prevention initiatives (Suter and Bruns 2009). Such ecologically informed approaches may disrupt the cascade from environmental adversity to EF deficits and substance use at multiple points.

### 4.6 Limitations and Future Directions

Several limitations inform future research. Although providing temporal ordering, our design assessed ACEs retrospectively as dichotomous indicators, omitting severity and timing (McLaughlin et al. 2014). Similarly, EF relied solely on caregiver report, which captures constructs distinct from performance tasks (Toplak et al. 2013; Trossman et al. 2022). Future studies should utilize dimensional ACE measures and multi‐informant assessments distinguishing “cold” from “hot” EF. Relatedly, baseline EF could not be statistically controlled, as the BDEFS‐CA was first administered at the year‐3 wave of the ABCD Study; this limits our ability to account for pre‐existing EF deficits that may have been present prior to the ACE assessments.

In addition, ACE exposure was captured only through the baseline and Year‐1 assessments (ages 9–11), rather than the full developmental window through age 18 used in standard ACE research (Felitti et al. 1998; Grummitt et al. 2022; Hughes et al. 2017). We restricted ACE measurement to these earliest assessments for two reasons. First, prospective mediation requires the predictor to unambiguously precede the mediator (Year‐3 EF) and outcome (Year‐4 substance use). Second, emerging adolescent EF and behaviors can evoke adversities like bullying and family conflict through transactional processes (Holmes et al. 2016; Jaffee and Price 2007). Including proximal exposures, therefore, risks conflating ACEs as developmental antecedents with ACEs as downstream consequences of existing self‐regulation. Nevertheless, this restricted window likely suppresses our sample mean (M = 2.23) and misses later‐emerging adversities that could exert independent effects. Future work using subsequent ABCD waves should extend ACE measurement using bidirectional analytic approaches. Additionally, sensitive ACEs such as sexual abuse are subject to substantial underreporting, particularly among children and early adolescents (Alaggia et al. 2019; Lemaigre et al. 2017). The very low endorsement rate of sexual abuse in our sample (0.9%) is therefore likely an underestimate, suggesting that some affected youth were misclassified as unexposed.

Furthermore, despite extensive covariates, residual confounding by unmeasured factors (e.g., parenting quality, genetic liability) remains possible (Grummitt et al. 2022), and multiple mediation tests increase Type I error risk. Future research could employ parallel‐mediator models testing EF against mechanisms like emotion dysregulation to explain direct effects such as that of sexual abuse (Grummitt et al. 2021), or use data‐driven approaches to empirically derive dimensions like unpredictability (E. P. Davis, Stout, et al. 2017). Finally, longer‐term designs are needed to model substance use escalation trajectories and reciprocal “neurotoxicity” effects on subsequent EF (Hoffmann and Jones 2022). In addition, although the missing Year‐4 substance use data primarily reflected the rolling structure of the ABCD Study's data releases rather than selective participant attrition, our analyses were necessarily restricted to participants with complete outcome data, and sensitivity analyses indicated small but statistically significant differences in ACE exposure between included and excluded participants. While these effect sizes were negligible in magnitude, future work using later ABCD releases with more complete Year‐4 data could provide a more comprehensive test of these associations.

### 4.7 Conclusion

This study provides prospective evidence that daily‐life EF deficits are a significant pathway linking cumulative childhood adversity to early adolescent substance use. The findings support a vulnerability model of addiction and offer a nuanced view of the DMAP. While the EF pathway is robust for deprivation‐based and many chaotic threat‐based adversities, it is bypassed by sexual abuse, which retains a direct effect, suggesting its impact operates through a distinct, trauma‐specific pathway. This highlights EF as a modifiable intervention target for many at‐risk youth but underscores the necessity of prioritizing distinct, trauma‐specific interventions for survivors of sexual abuse.

## Author Contributions

**Mahsa P. Yousefkhani:** conceptualization, methodology, data curation, investigation, formal analysis, visualization, project administration, writing – original draft, writing – review and editing. **Xiafei Wang:** conceptualization, methodology, funding acquisition, writing – review and editing. **Sara A. Vasilenko:** supervision, conceptualization, methodology, validation, funding acquisition, resources, writing – review and editing.

## Ethics Statement

The ABCD study was reviewed by the central institutional review board (IRB) at the University of California, San Diego, as well as local IRBs at individual sites. Written informed consent (parents) and assent (minor children) were obtained for all participants.

## Conflicts of Interest

The authors declare no conflicts of interest.

## Data Availability

Data are from the Adolescent Brain and Cognitive Development Study (https://abcdstudy.org). Data are available to researchers through the National Institute of Mental Health Data Archive, and investigators can apply for access at https://nda.nih.gov/.
